# Managed care and inpatient mortality in adults: effect of primary payer

**DOI:** 10.1186/s12913-017-2062-1

**Published:** 2017-02-08

**Authors:** Anika L. Hines, Susan O. Raetzman, Marguerite L. Barrett, Ernest Moy, Roxanne M. Andrews

**Affiliations:** 1 Truven Health Analytics, 7700 Old Georgetown Road, Bethesda, 20814 MD USA; 2 Johns Hopkins School of Medicine, Baltimore, MD USA; 3 ML Barrett, Inc, Del Mar, CA USA; 4 Agency for Healthcare Research and Quality, Rockville, MD USA; 5 National Center for Health Statistics, Hyattsville, MD USA

**Keywords:** Managed care, Inpatient mortality, Fee for service

## Abstract

**Background:**

Because managed care is increasingly prevalent in health care finance and delivery, it is important to ascertain its effects on health care quality relative to that of fee-for-service plans. Some stakeholders are concerned that basing gatekeeping, provider selection, and utilization management on cost may lower quality of care. To date, research on this topic has been inconclusive, largely because of variation in research methods and covariates. Patient age has been the only consistently evaluated outcome predictor. This study provides a comprehensive assessment of the association between managed care and inpatient mortality for Medicare and privately insured patients.

**Methods:**

A cross-sectional design was used to examine the association between managed care and inpatient mortality for four common inpatient conditions. Data from the 2009 Healthcare Cost and Utilization Project State Inpatient Databases for 11 states were linked to data from the American Hospital Association Annual Survey Database. Hospital discharges were categorized as managed care or fee for service. A phased approach to multivariate logistic modeling examined the likelihood of inpatient mortality when adjusting for individual patient and hospital characteristics and for county fixed effects.

**Results:**

Results showed different effects of managed care for Medicare and privately insured patients. Privately insured patients in managed care had an advantage over their fee-for-service counterparts in inpatient mortality for acute myocardial infarction, stroke, pneumonia, and congestive heart failure; no such advantage was found for the Medicare managed care population. To the extent that the study showed a protective effect of privately insured managed care, it was driven by individuals aged 65 years and older, who had consistently better outcomes than their non-managed care counterparts.

**Conclusions:**

Privately insured patients in managed care plans, especially older adults, had better outcomes than those in fee-for-service plans. Patients in Medicare managed care had outcomes similar to those in Medicare FFS. Additional research is needed to understand the role of patient selection, hospital quality, and differences among county populations in the decreased odds of inpatient mortality among patients in private managed care and to determine why this result does not hold for Medicare.

## Background

The emergence of managed care in health care finance and delivery has created a need to evaluate whether it improves or erodes health care quality compared with fee-for-service plans and to establish which factors contribute to any differences in outcomes. Some stakeholders have been concerned that implementation of gatekeeping, constraints on provider selection, and utilization management based on cost might contribute to reduced quality of care. Unfortunately, it is difficult to draw conclusions about differential outcomes in managed care versus fee-for-service plans from the literature. Direct comparisons are problematic because individual investigations vary in research methods and covariates. Additionally, effects may be masked if managed care attracts healthier patients who accept less personal control over specific provider and service choices in exchange for lower premiums.

An additional layer of contention in the managed care debate involves the health care outcomes of those insured by Medicare versus private insurance. Overall, inpatient mortality has steadily decreased over time [1–3]. One recent study of observed rates of inpatient mortality suggested that mortality may be declining more rapidly for Medicare patients compared with privately insured patients for acute myocardial infarction (AMI), stroke, pneumonia, and congestive heart failure (CHF) [3].

Research findings on the association between managed care and inpatient mortality for Medicare and privately insured patients have been mixed. Two studies that compared Medicare beneficiaries in managed care and fee-for-service settings found no differences in inpatient mortality [4, 5]. However, these studies examined patients hospitalized for only one medical condition. In a study of Medicare beneficiaries only, Afendulis and colleagues [6] found that patients in Medicare Advantage had fewer hospitalizations and lower mortality than those in traditional Medicare, but they concluded that these differences may be attributable to higher payment rates for more services. Additional studies included all payers and found that patients in managed care had lower inpatient mortality rates compared with patients in fee-for-service plans [7, 8]. However, one of these studies was limited to intensive care unit data in a single state, and the other study examined a single diagnosis-related group.

Although authors have cited results from studies with similar findings to strengthen the discussion of their own work, the research designs have not always been comparable. Studies have reported that patient characteristics such as age, sex, payer, and severity of illness influence the association between managed care and inpatient mortality [5, 7, 8]. Fewer studies have evaluated the contribution of hospital characteristics to this relationship [8]. With the exception of age, no patient or hospital predictor has been included consistently across the studies. Thus, questions remain regarding the effects of patient and hospital characteristics on the inpatient mortality of patients in managed care.

The purpose of this study was to provide a comprehensive assessment of the association between managed care and inpatient mortality among Medicare and privately insured patients with four common inpatient conditions. We made adjustments for patient characteristics, hospital characteristics, and unobserved county effects. We used recent data from a population of patients from 11 states. Further, we examined managed care within the context of Medicare and private insurance environments to determine whether expected primary payer modifies this relationship.

## Methods

### Data source

We used the 2009 Healthcare Cost and Utilization Project (HCUP) State Inpatient Databases (SID). HCUP is a family of health care databases developed through a voluntary federal-state-industry partnership sponsored by the Agency for Healthcare Research and Quality. The SID include a census of hospitals for states with a summary record for each discharge, regardless of payer. This analysis included inpatient discharges for both Medicare and privately insured patients aged 18 years and older from nonfederal, community, nonrehabilitation hospitals. Patients who were transferred out to another acute care hospital were excluded from the analysis, whereas patients who were transferred in to the hospital were included. Eleven states reported expected primary payer categories that distinguished between managed care and non-managed care plans: Arizona, California, Connecticut, Massachusetts, Michigan, Minnesota, Nevada, New Hampshire, New York, Ohio, and Pennsylvania. These states captured 36% of total adult (18 years and older) U.S. discharges and 38% of the adult U.S. population in 2009. We linked SID data to the American Hospital Association (AHA) Annual Survey Database to identify hospital characteristics. The HCUP databases are consistent with the definition of limited data sets under the Health Insurance Portability and Accountability Act (HIPAA) Privacy Rule and contain no direct patient identifiers. The use of HCUP data is not considered human subjects research by the Agency for Healthcare Research and Quality institutional review board.

### Data categorization

We categorized each discharge as managed care or fee-for-service on the basis of the expected primary payer coding. Six of the 11 states reported categories coded as *health maintenance organization (HMO)*; the other states reported either a *managed care* category or an *HMO and managed care* category. For the purpose of this study, we categorized discharges coded as *HMO, managed care,* or *HMO and managed care* by states as managed care. This broad term reflects the heterogeneity in reporting among states. We categorized as fee-for-service all discharges not explicitly identified in the state data as managed care as defined above. We further stratified managed care categories by Medicare and private insurance to discern any modifying effects of these distinctive groups.

### Outcome measures

#### Inpatient mortality

The primary outcome for this analysis was in-hospital mortality for four high-volume conditions: AMI, stroke, pneumonia, and CHF. We selected these conditions because of their prevalence among hospital discharges, which boosts statistical power to detect small differences. The mortality outcome for the regressions was defined dichotomously—whether a patient died in the hospital (*Yes* or *No*) based on the discharge disposition.

#### Patient and hospital characteristics

We linked patient data elements from the SID to hospital elements from the AHA database to describe the study population and to evaluate the characteristics as covariates or modifiers in the regression model. Patient characteristics included age, sex, All Patient Refined Diagnosis-Related Group (APR-DRG) and the associated risk of mortality subclass, and median household income of the patient’s residential ZIP Code (in quartiles). Consistent with other studies of inpatient mortality [9], we included this variable as the best available proxy of the patient’s income and purchasing choices. Hospital characteristics included the number of hospital beds, teaching status, ownership, and urban/rural location. We classified urban/rural locations of hospitals on the basis of the scheme for U.S. counties developed for the National Center for Health Statistics (NCHS) [10]. We excluded managed care penetration as a covariate in the analysis on the basis of findings of previous studies that ruled out its role as a predictor of the outcome of interest [7].

#### Hospital fixed effects

To better understand the impact of unobservable hospital-level factors related to quality of care, we examined hospital fixed effects as covariates in a separate model including patient characteristics and county fixed effects. We included dummy variables for individual hospitals visited by patients.

#### Geographic fixed effects

We also examined county fixed effects as covariates. Dowd and colleagues [11] found that estimated overall mortality differences between managed care and fee-for-service patients were sensitive to geographic fixed effects. Although we did not expect inpatient mortality to be strongly affected by county characteristics (as would be expected with rates of population mortality that may be driven by underlying county-level characteristics, such as availability of resources), we included dummy variables for the county locations of the patients’ residences. These inclusions controlled for other “unobservable” factors that could not be measured directly.

### Data analyses

We used SAS (SAS Institute, Inc; Cary, NC) statistical software Version 9.2 to perform statistical analyses. We identified patients treated for AMI, stroke, pneumonia, and CHF on the basis of specifications of the denominator in corresponding Inpatient Quality Indicators (IQIs) [12]. The IQIs are measures of inpatient quality endorsed by the National Quality Forum that use readily available administrative data. We then used multivariate logistic modeling to examine the likelihood of dying in the hospital, adjusting for patient, hospital, and county factors. For each condition, we performed separate logistic regressions for Medicare and private insurance.

We used a phased approach to examine the contributions of patient and hospital characteristics to the relationship between managed care status and inpatient mortality. We began with an unadjusted model of the association between managed care status and mortality. In subsequent models, we added patient characteristics followed by patient characteristics plus hospital characteristics. We then ran separate models that included individual patient characteristics plus hospital fixed effects to adjust for unobservable hospital characteristics. Lastly, we ran models that included patient characteristics, hospital characteristics, and county fixed effects. Several of the models with either hospital fixed effects or county fixed effects did not converge. Detailed tables with the results of full multivariate models are included in the Appendix.

### Sensitivity analysis

Our categorization of managed care is based on codes used by statewide data organizations, and these codes are not consistently defined. This variation in coding could create some bias. In our groupings of managed care versus fee-for-service, we assumed that a limited number of categories encompassed managed care on the basis of the labeling provided by states. It is possible that some managed care groups were included as fee-for-service and vice versa. Although we used the most stringent classification approach available, some of this bias is unavoidable because of the nature of the data and collection methods. Consequently, a lack of distinction between these groups could dilute any potential differences between individuals in managed care versus fee-for-service. We address this limitation in a sensitivity analysis of fewer states with more stringently defined HMO categories.

## Results

### Demographic characteristics

Table 1 contains the demographic characteristics of patients with AMI, stroke, pneumonia, and CHF in all plan types and the facilities from which they were discharged. Compared with Medicare patients in non-managed care, patients in Medicare managed care were slightly older, resided in higher median income ZIP Code areas, and were more likely to have been discharged from hospitals in large central metropolitan areas, teaching hospitals, and hospitals with 300 or more beds. The Medicare managed care population also was less likely than their non-managed care counterparts to have congestive heart failure, chronic pulmonary disease, diabetes with complications, and depression.

**Table 1:** Demographic and hospital characteristics of populations in Medicare and private insurance, 2009

Characteristic^a,b^	Medicare managed care (*n* = 168,700)		Medicare fee for service (*n* = 562,610)			Private managed care (*n* = 84,170)		Private fee for service (*n* = 115,244)		
	Mean, %	SE	Mean, %	SE	*p*	Mean, %	SE	Mean, %	SE	*p*
Age in years, mean	78.04	0.02	77.43	0.02	*	57.98	0.05	57.96	0.04	
Sex, %										
Female	52.33	0.12	53.51	0.07	*	41.39	0.17	39.78	0.15	*
Median household income by ZIP Code, %										
Lowest (<$39,999)	22.61	0.10	22.70	0.06		18.30	0.13	19.06	0.12	*
Low ($40,000-$49,999)	24.10	0.10	26.42	0.06	*	21.93	0.14	26.58	0.13	*
Moderate ($50,000-$65,999)	26.41	0.11	26.03	0.06	*	28.20	0.16	27.08	0.13	*
High (>$66,000)	26.88	0.11	24.85	0.06	*	31.56	0.16	27.28	0.13	*
Comorbidities										
Congestive heart failure	10.82	0.08	11.89	0.04	*	5.19	0.08	4.90	0.06	*
Chronic pulmonary disease	32.14	0.11	34.52	0.06	*	24.07	0.15	24.89	0.13	*
Hypertension	70.49	0.11	67.61	0.06	*	59.03	0.17	56.11	0.15	*
Peripheral vascular disease	11.44	0.08	10.18	0.04	*	6.00	0.08	5.64	0.07	*
Diabetes with chronic complications	25.99	0.11	28.17	0.06	*	23.20	0.15	23.67	0.13	*
Diabetes without chronic complications	10.21	0.07	7.17	0.03	*	7.89	0.09	5.15	0.07	*
Hypothyroidism	15.40	0.09	15.80	0.05	*	8.52	0.10	8.82	0.08	*
Renal failure	27.76	0.11	27.78	0.06		14.44	0.12	12.28	0.10	*
Fluid and electrolyte disorders	24.49	0.11	27.87	0.06	*	21.71	0.14	22.30	0.12	*
Obesity	8.07	0.07	8.10	0.04		15.61	0.13	14.20	0.10	*
Deficiency anemias	23.24	0.10	24.99	0.06	*	16.52	0.13	14.09	0.10	*
Depression	8.01	0.07	9.49	0.04	*	8.40	0.10	8.51	0.08	
Hospital location, %										
Large central metropolitan	53.77	0.12	37.87	0.07	*	57.58	0.17	36.78	0.14	*
Large fringe metropolitan	19.88	0.10	19.34	0.05	*	17.90	0.13	20.44	0.12	*
Medium metropolitan	18.47	0.10	23.81	0.06	*	18.34	0.13	25.83	0.13	*
Small metropolitan	3.15	0.04	6.96	0.03	*	1.97	0.05	6.46	0.07	*
Micropolitan	3.78	0.05	9.42	0.04	*	3.14	0.06	8.67	0.08	*
Not metropolitan or micropolitan	0.95	0.02	2.60	0.02	*	1.08	0.04	1.82	0.04	*
Hospital ownership, %										
Government	6.13	0.06	7.25	0.03	*	5.85	0.08	7.05	0.08	*
Private, not-for-profit	87.55	0.08	86.07	0.05	*	86.26	0.12	87.93	0.10	*
Private, for-profit	6.32	0.06	6.68	0.03	*	7.89	0.09	5.01	0.06	*
Hospital teaching, %										
Teaching	46.25	0.12	37.35	0.07	*	46.47	0.17	43.48	0.15	*
Number of hospital beds, %										
< 100	6.58	0.06	11.76	0.04	*	6.33	0.08	8.79	0.08	*
100-299	37.97	0.12	38.75	0.07	*	35.44	0.17	36.18	0.14	*
300-499	32.91	0.12	28.28	0.06	*	33.13	0.16	28.69	0.13	*
500+	22.54	0.10	21.21	0.05	*	25.10	0.15	26.34	0.13	*

Abbreviation: *SE*, standard error

^a^Patient characteristics were age, sex, community income, and All Patient Refined-Diagnosis Related Group (APR-DRG)

^b^Hospital characteristics were urban/rural location, ownership, teaching status, and bed size

*p < 0.05

Source: Agency for Healthcare Research and Quality, Center for Delivery, Organization, and Markets, Healthcare Cost and Utilization Project, State Inpatient Databases, 2009, from the following 11 states: Arizona, California, Connecticut, Massachusetts, Michigan, Minnesota, New Hampshire, Nevada, New York, Ohio, and Pennsylvania

Patients in private managed care were similar in age to their counterparts in non-managed care, but the private managed care group had a greater percentage of women and individuals residing in ZIP Codes with median household incomes greater than $50,000. In addition, compared with their non-managed care counterparts, a greater percentage of patients in private managed care were discharged from hospitals in large central metropolitan areas, private for-profit hospitals, teaching hospitals, and hospitals with 300 to 499 beds.

### Observed rates of inpatient mortality by insurance type

**Fig. 1:**
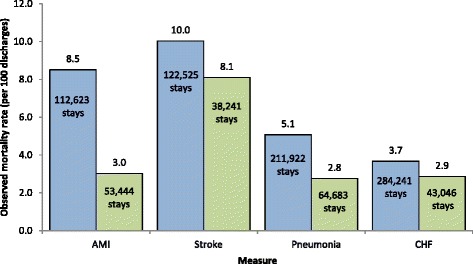
Observed inpatient mortality rates for AMI, stroke, pneumonia, and CHF for patients in Medicare and private insurance, 2009. Legend: *Blue bars* indicate Medicare patients; *green bars* indicate private insured patients. Abbreviations: AMI, acute myocardial infarction; CHF, congestive heart failure. Source: Agency for Healthcare Research and Quality, Center for Delivery, Organization, and Markets, Healthcare Cost and Utilization Project, State Inpatient Databases, 2009, from the following 11 states: Arizona, California, Connecticut, Massachusetts, Michigan, Minnesota, New Hampshire, Nevada, New York, Ohio, and Pennsylvania

Figure 1 displays observed rates of inpatient mortality for each of the four conditions of interest by insurance type. Compared with private insurance, patients with Medicare had higher rates of inpatient mortality for all four conditions. For AMI, the Medicare inpatient mortality rate was nearly three times that of the privately insured—the largest difference in rates across conditions.

### Controlling for patient, hospital, and county characteristics

Table 2 shows results from models of inpatient mortality for patients with Medicare and private insurance, comparing managed care with fee-for-service plans. Although patients in Medicare managed care plans had lower odds of inpatient death for stroke and CHF in models controlling for patient characteristics, these differences disappeared when hospital characteristics or hospital fixed effects were added to the model, and they remained insignificant when county fixed effects were added (Table 2).

**Table 2:** Inpatient mortality for patients with Medicare and private insurance, comparing managed care to fee-for-service plans, 2009

Measure	Sample size for managed care and FFS	Patient characteristics^a^			Patient + hospital characteristics^b^			Patient characteristics + hospital fixed effects			Patient + hospital characteristics + county fixed effects		
		OR	95% CI	Difference^c^	OR	95% CI	Difference^c^	OR	95% CI	Difference^c^	OR	95% CI	Difference^c^
Medicare managed care vs. Medicare FFS													
AMI	112,623	0.97	0.92, 1.02		0.98	0.93, 1.04		0.98	0.92, 1.04		0.98	0.93, 1.04	
Stroke	122,525	0.93	0.89, 0.98	↓	0.98	0.93, 1.03		0.97	0.91, 1.03		0.98	0.93, 1.03	
Pneumonia	211,921	1.03	0.98, 1.09		1.07	1.02, 1.13	↑	0.99	0.93, 1.05		1.05	0.99, 1.11	
CHF	284,241	0.95	0.90, 0.99	↓	0.98	0.93, 1.03		<did not converge>			0.95	0.90, 1.00	
Private managed care vs. private FFS													
AMI	53,444	0.87	0.77, 0.97	↓	0.88	0.78, 0.98	↓	<did not converge>			0.86	0.76, 0.98	↓
Stroke	38,241	0.76	0.69, 0.83	↓	0.80	0.73, 0.87	↓	0.84	0.75, 0.94	↓	0.79	0.71, 0.87	↓
Pneumonia	64,683	0.90	0.82, 1.00		0.89	0.80, 0.99	↓	0.83	0.72, 0.95	↓	0.88	0.78, 0.98	↓
CHF	43,046	0.62	0.55, 0.70	↓	0.64	0.57, 0.73	↓	<did not converge>			<did not converge>		

Abbreviations: *AMI*, acute myocardial infarction; *CHF*, congestive heart failure; *CI*, confidence interval; *FFS*, fee for service; *OR*, odds ratio

^a^Patient characteristics were age, sex, All Patient Refined-Diagnosis Related Group (APR-DRG), and community income

^b^Hospital characteristics were bed size, ownership, teaching status, and urban/rural location

^c^A down arrow indicates the mortality rate for managed care is significantly lower than FFS at *p* < 0.05. An up arrow indicates the mortality rate for managed care is significantly higher than FFS at *p* < 0.05

Source: Agency for Healthcare Research and Quality, Center for Delivery, Organization, and Markets, Healthcare Cost and Utilization Project, State Inpatient Databases, 2009, from the following 11 states: Arizona, California, Connecticut, Massachusetts, Michigan, Minnesota, New Hampshire, Nevada, New York, Ohio, and Pennsylvania

Among privately insured patients, the association between managed care and inpatient mortality was consistently negative and typically statistically significant across conditions. Patients in private managed care plans had lower odds of inpatient mortality for all four conditions when controlling for patient and hospital characteristics. Managed care was particularly protective among patients with private insurance and CHF (36% lower odds of mortality) or stroke (20% lower odds of mortality). The addition of county fixed effects to the models strengthened the managed care effects for AMI, stroke, and pneumonia.

To assess potential modifying effects of age among the privately insured, we ran additional logistic models for individuals younger than 65 years and for those 65 years and older (Table 3).

**Table 3:** Inpatient mortality for patients with private insurance, comparing managed care to fee-for-service plans, by patient age, 2009

Measure	Sample size for managed care and FFS	Patient characteristics^a^			Patient + hospital characteristics^b^			Patient characteristics + hospital fixed effects			Patient + hospital characteristics + county fixed effects		
		OR	95% CI	Difference^c^	OR	95% CI	Difference^c^	OR	95% CI	Difference^c^	OR	95% CI	Difference^c^
Private managed care vs. private FFS, age <65 years													
AMI	44,580	0.91	0.78, 1.05		0.91	0.79, 1.06		0.89	0.75, 1.06		0.89	0.75, 1.05	
Stroke	28,713	0.87	0.77, 0.97	↓	0.90	0.80, 1.01		0.89	0.78, 1.01		0.87	0.77, 0.99	↓
Pneumonia	51,636	1.05	0.92, 1.20		1.02	0.90, 1.17		1.00	0.85, 1.17		1.01	0.88, 1.17	
CHF	26,980	0.84	0.69, 1.03		0.81	0.66, 0.99	↓	0.83	0.66, 1.04		0.75	0.60, 0.94	↓
Private managed care vs. private FFS, age ≥65 years													
AMI	8,864	0.80	0.67, 0.95	↓	0.82	0.69, 0.98	↓	<did not converge>			<did not converge>		
Stroke	9,528	0.64	0.55, 0.73	↓	0.70	0.60, 0.81	↓	<did not converge>			<did not converge>		
Pneumonia	13,047	0.73	0.62, 0.86	↓	0.73	0.62, 0.86	↓	<did not converge>			<did not converge>		
CHF	16,066	0.52	0.45, 0.61	↓	0.56	0.47, 0.66	↓	<did not converge>			<did not converge>		

Abbreviations: *AMI*, acute myocardial infarction; *CHF*, congestive heart failure; *CI*, confidence interval; *FFS*, fee for service; *OR*, odds ratio

^a^Patient characteristics were age, sex, All Patient Refined-Diagnosis Related Group (APR-DRG), and community income

^b^Hospital characteristics were bed size, ownership, teaching status, and urban/rural location

^c^A down arrow indicates the mortality rate for managed care is significantly lower than FFS at *p* < 0.05. An up arrow indicates the mortality rate for managed care is significantly higher than FFS at *p* < 0.05

Source: Agency for Healthcare Research and Quality, Center for Delivery, Organization, and Markets, Healthcare Cost and Utilization Project, State Inpatient Databases, 2009, from the following 11 states: Arizona, California, Connecticut, Massachusetts, Michigan, Minnesota, New Hampshire, Nevada, New York, Ohio, and Pennsylvania

In the privately insured population aged 65 years and older, managed care was negatively associated with inpatient mortality for all four conditions when controlling for patient and hospital characteristics. The models including either hospital fixed effects or county fixed effects failed to converge, likely because of the small sample size of the group aged 65 years and older relative to the large number of possible hospitals and counties represented. Patients who were privately insured and younger than 65 years demonstrated inconsistent results across conditions. There were no differences in inpatient mortality for younger patients with AMI or pneumonia in private managed care and fee-for-service plans, but outcomes favored managed care for stroke and CHF when controlling for patient characteristics, hospital characteristics, and county fixed effects.

To assess how a stricter definition would affect our findings, we performed a sensitivity analysis using three states (California, New York, and Pennsylvania) with managed care defined by primary payer categories that were explicitly named *HMO* (Table 4). Compared with the main analysis, this sensitivity analysis has much smaller sample sizes and less geographic diversity.

**Table 4:** Inpatient mortality for patients with Medicare and private insurance, comparing managed care to fee-for-service plans using a stringent definition of health maintenance organization, 2009

Measure	Sample size for managed care and FFS	Patient characteristics^a^			Patient + hospital characteristics^b^			Patient characteristics + hospital fixed effects			Patient + hospital characteristics + county fixed effects		
		OR	95% CI	Difference^c^	OR	95% CI	Difference^c^	OR	95% CI	Difference^c^	OR	95% CI	Difference^c^
Medicare managed care vs. Medicare FFS													
AMI	61,159	0.97	0.91, 1.04		0.98	0.91, 1.04		1.01	0.94, 1.09		1.00	0.94, 1.08	
Stroke	69,803	0.91	0.86, 0.97	↓	0.96	0.90, 1.03		0.99	0.92, 1.06		0.98	0.92, 1.05	
Pneumonia	114,515	0.99	0.94, 1.06		1.03	0.97, 1.09		0.99	0.92, 1.06		1.05	0.98, 1.12	
CHF	157,794	0.90	0.84, 0.95	↓	0.91	0.86, 0.97	↓	<did not converge>			0.93	0.87, 0.99	↓
Private managed care vs. private FFS													
AMI	27,577	0.86	0.74, 1.00		0.88	0.75, 1.02		<did not converge>			0.88	0.74, 1.05	
Stroke	21,510	0.87	0.78, 0.98	↓	0.88	0.78, 0.98	↓	1.02	0.88, 1.18		0.93	0.82, 1.07	
Pneumonia	33,573	0.95	0.83, 1.08		0.92	0.80, 1.05		0.96	0.80, 1.14		0.93	0.80, 1.08	
CHF	22,926	0.66	0.56, 0.78	↓	0.67	0.56, 0.79	↓	<did not converge>			<did not converge>		

Abbreviations: *AMI*, acute myocardial infarction; *CHF*, congestive heart failure; *CI*, confidence interval; *FFS*, fee for service; *OR*, odds ratio

^a^Patient characteristics were age, sex, All Patient Refined-Diagnosis Related Group (APR-DRG), and community income

^b^Hospital characteristics were bed size, ownership, teaching status, and urban/rural location

^c^A down arrow indicates the mortality rate for managed care is significantly lower than FFS at *p* < 0.05. An up arrow indicates the mortality rate for managed care is significantly higher than FFS at *p* < 0.05

Source: Agency for Healthcare Research and Quality, Center for Delivery, Organization, and Markets, Healthcare Cost and Utilization Project, State Inpatient Databases, 2009, from the following 3 states: California, New York, and Pennsylvania

We found similar results favoring managed care among privately insured patients with stroke and CHF when controlling for patient and hospital characteristics, but there were no differences in outcomes between patients with AMI and pneumonia in managed care versus fee-for-service plans. Patients with Medicare managed care had lower odds of inpatient mortality for CHF than did patients with Medicare fee-for-service plans.

## Discussion

For Medicare beneficiaries, outcomes differed by condition, particularly when hospital characteristics were taken into account. These results confirm those of Carlisle and colleagues [4] and Smith and colleagues [5], who also found that Medicare managed care was not related to AMI and stroke mortality outcomes. Moreover, the phased approach of this analysis revealed the unique contributions of hospital characteristics to mortality outcomes among patients in Medicare managed care. For example, although there were no differences in the outcomes of patients with pneumonia in managed care and fee-for-service Medicare when controlling for patient characteristics, a closer look at the detailed hospital model (Appendix Table 9) revealed that Medicare patients with pneumonia who were admitted to specific types of hospitals—those that were government-owned, had smaller bed sizes, and were in nonmetropolitan areas—demonstrated higher odds of mortality than similar patients admitted to larger, urban, privately owned hospitals. A previous study revealed that the Medicare Advantage population was treated more often in facilities with lower resource cost and higher risk-adjusted mortality relative to patients in fee-for-service plans [13]. Limited resources associated with hospitals in smaller geographic areas [14] may affect health care quality and outcomes for patients with pneumonia in Medicare who are treated in these types of facilities.

Among privately insured patients, those in managed care demonstrated lower rates of inpatient mortality for all four conditions after adjusting for other patient and hospital characteristics. Older age and the severity of the patient’s condition are powerful predictors of inpatient mortality, but they do not explain why managed care is associated with lower odds of inpatient mortality in this population. Despite the adjustments for patient characteristics and clinical factors (including APR-DRG severity of disease and associated risk of mortality subclass), the privately insured managed care population had lower odds of inpatient mortality. Interestingly, patients in privately insured managed care plans also demonstrated higher rates of certain common comorbidities (i.e., CHF, diabetes without chronic complications, renal failure, and obesity) than their fee-for-service counterparts. Similar to the experience of Medicare patients, hospital characteristics were strong predictors of inpatient mortality among privately insured patients. Whether patients in privately insured managed care plans systematically visit better quality hospitals than their fee-for-service counterparts is a topic worthy of future study. Furthermore, the study of the interactions between managed care and hospital characteristics as predictors could illuminate the mechanism through which managed care influences inpatient mortality.

An additional contribution of this work is the detailed examination of mortality outcomes among patients with private managed care; previous studies have focused on Medicare [4, 5]. We found that the privately insured population aged 65 years and older drove favorable managed care outcomes across the conditions studied. Although the sample sizes precluded our analysis of county fixed effects for this group, patients aged 65 years and older in managed care demonstrated lower rates of inpatient mortality compared with their fee-for-service counterparts for all four conditions. The protective effect of managed care was stronger for patients aged 65 years and older with private insurance than for their younger counterparts. There was no such age effect for Medicare outcomes when comparing beneficiaries aged 65 years and older to those younger than 65 years (data not shown). One explanation could be that privately insured individuals aged 65 years and older often are still employed or may have more wealth than those for whom Medicare is the primary payer. Either of these factors could be associated with better baseline health status, which could confound the likelihood of death from any of these conditions. Our data indicate that a higher share of patients in private managed care than in Medicare managed care were in the higher income quartiles. However, counter to this possible explanation, Appendix Tables 5–12 show that income was not a statistically significant contributor among models in this study. Therefore, additional investigation is needed to understand the potentially protective effect of managed care in the private sector for those aged 65 years and older, and the interpretation of these findings should be treated cautiously.

Variations in outcomes between patients in Medicare and private managed care relative to their fee-for-service counterparts bring into question differences in managed care experiences by payer. Are patients who are in private managed care treated in better hospitals than patients in Medicare managed care? Our limited descriptive information regarding hospitals from which these two groups were discharged showed similar distributions with regard to ownership, teaching status, and bed size. However, these characteristics do not fully capture the quality of care delivered. Selective contracting with hospitals, or the practice of contracting with certain providers to ensure quality or to contain costs, has previously been studied as influencing managed care and patient outcomes. This practice is not likely to be the primary driver of differences between the outcomes of privately insured managed care and fee-for-service populations [15]. However, the ways in which selective contracting or other managed care mechanisms might favor private insurance over Medicare are not known. Analysis of hospital fixed effects using an indicator for each hospital demonstrated results similar to the models that controlled for individual hospital factors. Future research should continue to explore the quality of care delivered at hospitals chosen by patients in private managed care and those to which they are referred, especially for individuals aged 65 years and older. In addition, future studies should explore the association of managed care status with outcomes by severity class of condition to discern whether there is an insurance effect.

The findings of this study should be interpreted within the context of a few limitations. First, the cross-sectional approach of this study prohibited investigators from capturing the full episode of care preceding the inpatient admission. The lack of data on past medical history limits the risk adjustment for clinical factors included in the models to conditions reported on the current discharge record only. Therefore, we cannot discern whether inpatient death was more related to the current discharge or some previous care. Second, the HCUP SID only include information on in-hospital mortality. Therefore, post-discharge deaths are not included, leading to an underestimation of *overall* mortality for these conditions.

## Conclusions

We used hospital administrative data to examine the association between managed care and inpatient mortality, controlling for patient and hospital characteristics and county fixed effects. Although patients in private managed care had lower rates of inpatient mortality for AMI, stroke, pneumonia, and CHF compared with fee-for-service beneficiaries with hospitalizations for these conditions, patients in Medicare managed care did not experience decreased odds of mortality relative to their fee-for-service counterparts once hospital factors were controlled. Furthermore, although the advantage among patients in private managed care remained after controlling for patient and hospital characteristics as well as county fixed effects of the patient’s residence, the private managed care population aged 65 years and older drove the findings of protective effects of managed care with respect to inpatient mortality. Results of the hospital fixed effects models suggest that other unmeasured hospital factors may play a role in predicting inpatient mortality. Could the location of hospitals and availability of community resources drive these results across privately insured and Medicare patients under managed care? More research is needed to understand the relative roles of patient selection, hospital quality, and differences among county populations in decreased odds of inpatient mortality among patients in private managed care and the absence of that result among patients covered by Medicare.

## Appendix

**Table 5:** Association between Medicare managed care and inpatient mortality for acute myocardial infarction

Characteristic	Patient characteristics^a^			Patient + hospital characteristics^b^			Patient characteristic + hospital fixed effects			Patient + hospital characteristics + county fixed effects		
	Point estimate	95% Wald confidence limits		Point estimate	95% Wald confidence limits		Point estimate	95% Wald confidence limits		Point estimate	95% Wald confidence limits	
Managed care	0.969	0.919	1.021	0.982	0.931	1.036	0.979	0.922	1.039	0.983	0.929	1.040
Age 18–64 years	1.012	0.908	1.129	1.018	0.913	1.135	1.030	0.922	1.151	1.025	0.918	1.145
Age 65–74 years (REF)	REF	REF	REF	REF	REF	REF	REF	REF	REF	REF	REF	REF
Age 75–84 years	1.198	1.125	1.276	1.196	1.123	1.273	1.194	1.120	1.272	1.190	1.117	1.268
Age 85+ years	1.362	1.276	1.453	1.352	1.267	1.444	1.354	1.266	1.447	1.339	1.253	1.431
Male	REF	REF	REF	REF	REF	REF	REF	REF	REF	REF	REF	REF
Female	0.959	0.916	1.003	0.958	0.916	1.003	0.957	0.914	1.002	0.963	0.920	1.008
APRMORT_165002	0.332	0.144	0.768	0.339	0.147	0.783	0.343	0.148	0.793	0.334	0.144	0.771
APRMORT_165003	1.673	1.004	2.787	1.706	1.024	2.843	1.795	1.076	2.994	1.721	1.032	2.869
APRMORT_165004	16.335	10.790	24.728	16.742	11.058	25.347	18.402	12.135	27.905	17.412	11.487	26.392
APRMORT_174001	0.251	0.145	0.433	0.255	0.147	0.440	0.258	0.149	0.445	0.254	0.147	0.438
APRMORT_174002	0.823	0.532	1.274	0.837	0.541	1.295	0.862	0.557	1.335	0.838	0.541	1.297
APRMORT_174003	3.123	2.060	4.733	3.179	2.097	4.818	3.347	2.206	5.077	3.278	2.161	4.971
APRMORT_174004	33.770	22.759	50.108	34.426	23.199	51.087	38.038	25.605	56.509	36.594	24.639	54.349
APRMORT_190002	3.350	2.231	5.031	3.333	2.219	5.005	3.328	2.215	5.000	3.355	2.233	5.040
APRMORT_190003	8.975	6.061	13.290	8.898	6.009	13.177	9.044	6.103	13.401	9.090	6.135	13.468
APRMORT_190004	41.875	28.301	61.960	42.068	28.430	62.247	45.052	30.424	66.714	44.977	30.376	66.596
APRMORT_OTHER	12.857	8.627	19.162	13.113	8.797	19.545	13.865	9.292	20.689	13.436	9.008	20.041
Lowest income	1.021	0.956	1.089	1.003	0.937	1.073	0.991	0.913	1.075	1.010	0.928	1.099
Low income	1.049	0.986	1.115	1.031	0.967	1.100	1.037	0.961	1.119	1.060	0.980	1.146
Moderate income	0.997	0.938	1.061	0.998	0.938	1.062	0.982	0.915	1.054	0.997	0.929	1.069
High income	REF	REF	REF	REF	REF	REF	REF	REF	REF	REF	REF	REF
0-99 beds				1.168	1.062	1.284				1.185	1.067	1.315
100-299 beds				REF	REF	REF				REF	REF	REF
300-499 beds				1.009	0.952	1.070				1.019	0.953	1.090
500+ beds				1.029	0.954	1.109				1.066	0.979	1.160
Nonteaching				REF	REF	REF				REF	REF	REF
Teaching				0.931	0.878	0.988				0.908	0.846	0.975
Governmental				1.279	1.173	1.396				1.207	1.094	1.332
Not-for-profit				REF	REF	REF				REF	REF	REF
For-profit				0.995	0.908	1.092				0.971	0.873	1.081
Large metropolitan				REF	REF	REF						
Medium and small metropolitan				0.993	0.944	1.046						
Nonmetropolitan				1.104	1.008	1.209						

Abbreviation: *REF*, reference group

Notes: ^a^Patient characteristics were age, sex, All Patient Refined-Diagnosis Related Group (APR-DRG), and community income. ^b^Hospital characteristics were bed size, ownership, teaching status, and urban/rural location

Source: Agency for Healthcare Research and Quality, Center for Delivery, Organization, and Markets, Healthcare Cost and Utilization Project, State Inpatient Databases, 2009, from the following 11 states: Arizona, California, Connecticut, Massachusetts, Michigan, Minnesota, New Hampshire, Nevada, New York, Ohio, and Pennsylvania

**Table 6:** Association between private managed care and inpatient mortality for acute myocardial infarction

Characteristic	Patient characteristics^a^			Patient + hospital characteristics^b^			Patient characteristic + hospital fixed effects		Patient + hospital characteristics + county fixed effects		
	Point estimate	95% Wald confidence limits		Point estimate	95% Wald confidence limits		Point estimate	95% Wald confidence limits	Point estimate	95% Wald confidence limits	
Managed care	0.865	0.774	0.967	0.875	0.781	0.980	Failed to converge		0.861	0.758	0.979
Age 18–44 years	0.759	0.577	0.999	0.749	0.569	0.987			0.778	0.584	1.036
Age 45–64 years	REF	REF	REF	REF	REF	REF			REF	REF	REF
Age 65+ years	1.199	1.064	1.352	1.177	1.043	1.33			1.151	1.01	1.31
Male	REF	REF	REF	REF	REF	REF			REF	REF	REF
Female	1.087	0.969	1.221	1.081	0.963	1.214			1.122	0.994	1.266
APRMORT_165002	0.352	0.113	1.1	0.352	0.113	1.099			0.362	0.115	1.137
APRMORT_165003	3.616	1.958	6.677	3.62	1.96	6.686			3.554	1.9	6.648
APRMORT_165004	18.445	10.623	32.03	18.668	10.748	32.426			20.617	11.762	36.138
APRMORT_174001	0.095	0.04	0.221	0.094	0.04	0.221			0.091	0.039	0.214
APRMORT_174002	0.675	0.372	1.226	0.674	0.371	1.225			0.68	0.373	1.237
APRMORT_174003	5.988	3.495	10.256	5.994	3.499	10.27			6.221	3.612	10.712
APRMORT_174004	55.13	34.324	88.547	55.374	34.469	88.957			61.919	38.356	99.959
APRMORT_190002	4.869	2.899	8.178	4.847	2.886	8.141			5.085	3.016	8.572
APRMORT_190003	21.942	13.585	35.439	21.726	13.448	35.099			23.972	14.77	38.905
APRMORT_190004	122.158	76.067	196.178	122.164	76.063	196.208			144.677	89.571	233.684
APRMORT_OTHER	17.874	10.931	29.228	17.913	10.949	29.308			19.103	11.619	31.407
Lowest income	0.996	0.847	1.17	1.003	0.848	1.185			1.106	0.897	1.363
Low income	0.989	0.853	1.147	0.997	0.855	1.161			1.054	0.873	1.273
Moderate income	0.927	0.801	1.072	0.933	0.805	1.081			0.976	0.823	1.156
High income	REF	REF	REF	REF	REF	REF			REF	REF	REF
0-99 beds				1.061	0.796	1.414			1.11	0.792	1.555
100-299 beds				REF	REF	REF			REF	REF	REF
300-499 beds				1.079	0.934	1.245			1.063	0.899	1.256
500+ beds				1.224	1.017	1.474			1.243	1.001	1.544
Nonteaching				REF	REF	REF			REF	REF	REF
Teaching				0.802	0.693	0.929			0.776	0.648	0.928
Governmental				1.165	0.935	1.453			1.242	0.967	1.594
Not-for-profit				REF	REF	REF			REF	REF	REF
For-profit				0.801	0.637	1.007			0.705	0.541	0.92
Large metropolitan				REF	REF	REF					
Medium and small metropolitan				0.94	0.827	1.068					
Nonmetropolitan				1.072	0.837	1.372					

Abbreviation: *REF* indicates reference group

Notes: ^a^Patient characteristics were age, sex, All Patient Refined-Diagnosis Related Group (APR-DRG), and community income. ^b^Hospital characteristics were bed size, ownership, teaching status, and urban/rural location

Source: Agency for Healthcare Research and Quality, Center for Delivery, Organization, and Markets, Healthcare Cost and Utilization Project, State Inpatient Databases, 2009, from the following 11 states: Arizona, California, Connecticut, Massachusetts, Michigan, Minnesota, New Hampshire, Nevada, New York, Ohio, and Pennsylvania

**Table 7:** Association between Medicare managed care and inpatient mortality for stroke

Characteristic	Patient characteristics^a^			Patient + hospital characteristics^b^			Patient characteristic + hospital fixed effects			Patient + hospital characteristics + county fixed effects		
	Point estimate	95% Wald confidence limits		Point estimate	95% Wald confidence limits		Point estimate	95% Wald confidence limits		Point estimate	95% Wald confidence limits	
Managed care	0.931	0.885	0.98	0.978	0.929	1.029	0.969	0.914	1.028	0.979	0.927	1.034
Age 18–64 years	1.126	1.019	1.244	1.144	1.035	1.264	1.134	1.023	1.257	1.144	1.033	1.267
Age 65–74 years	REF	REF	REF	REF	REF	REF	REF	REF	REF	REF	REF	REF
Age 75–84 years	1.182	1.114	1.254	1.167	1.1	1.239	1.180	1.111	1.255	1.171	1.102	1.243
Age 85+ years	1.614	1.518	1.717	1.574	1.48	1.675	1.589	1.490	1.693	1.561	1.466	1.663
Male	REF	REF	REF	REF	REF	REF	REF	REF	REF	REF	REF	REF
Female	1.119	1.07	1.169	1.122	1.074	1.173	1.119	1.069	1.171	1.113	1.064	1.165
APRMORT_45002	4.166	3.283	5.286	4.184	3.297	5.31	4.207	3.313	5.341	4.219	3.324	5.356
APRMORT_45003	14.724	11.615	18.665	15.111	11.919	19.157	15.899	12.532	20.172	15.686	12.368	19.895
APRMORT_45004	98.22	77.642	124.25	103.991	82.182	131.59	117.459	92.720	148.798	112.299	88.691	142.192
APRMORT_44001	17.46	13.369	22.805	18.18	13.915	23.751	18.735	14.315	24.520	18.412	14.078	24.081
APRMORT_44002	25.718	20.201	32.743	26.697	20.964	33.998	26.618	20.881	33.932	26.749	20.992	34.085
APRMORT_44003	39.076	30.638	49.837	41.516	32.54	52.969	43.630	34.150	55.743	43.058	33.722	54.979
APRMORT_44004	378.362	298.54	479.52	409.913	323.26	519.8	485.194	381.913	616.405	453.1	356.958	575.137
APRMORT_21XXX	50.851	39.994	64.654	55.519	43.633	70.641	58.799	46.128	74.952	57.445	45.108	73.155
APRMORT_OTHER	22.104	17.3	28.241	23.958	18.742	30.626	25.232	19.713	32.297	24.529	19.177	31.374
Lowest income	0.854	0.802	0.91	0.803	0.753	0.857	0.883	0.816	0.957	0.94	0.868	1.019
Low income	0.883	0.833	0.937	0.815	0.766	0.867	0.922	0.857	0.993	0.953	0.884	1.026
Moderate income	0.944	0.891	1.000	0.916	0.864	0.971	1.000	0.935	1.069	1.025	0.959	1.094
High income	REF	REF	REF	REF	REF	REF	REF	REF	REF	REF	REF	REF
0-99 beds				1.251	1.134	1.38				1.348	1.212	1.499
100-299 beds				REF	REF	REF				REF	REF	REF
300-499 beds				0.961	0.907	1.019				1.022	0.957	1.091
500+ beds				1.054	0.982	1.131				1.026	0.948	1.11
Nonteaching				REF	REF	REF				REF	REF	REF
Teaching				0.862	0.814	0.912				0.86	0.804	0.92
Governmental				1.242	1.149	1.343				1.143	1.048	1.248
Not-for-profit				REF	REF	REF				REF	REF	REF
For-profit				0.799	0.725	0.88				0.776	0.693	0.868
Large metropolitan				REF	REF	REF						
Medium and small metropolitan				1.115	1.06	1.172						
Nonmetropolitan				1.504	1.366	1.656						

Abbreviation: *REF*, reference group

Notes: ^a^Patient characteristics were age, sex, All Patient Refined-Diagnosis Related Group (APR-DRG), and community income. ^b^Hospital characteristics were bed size, ownership, teaching status, and urban/rural location

Source: Agency for Healthcare Research and Quality, Center for Delivery, Organization, and Markets, Healthcare Cost and Utilization Project, State Inpatient Databases, 2009, from the following 11 states: Arizona, California, Connecticut, Massachusetts, Michigan, Minnesota, New Hampshire, Nevada, New York, Ohio, and Pennsylvania

**Table 8:** Association between private managed care and inpatient mortality for stroke

Characteristic	Patient characteristics^a^			Patient + hospital characteristics^b^			Patient characteristic + hospital fixed effects			Patient + hospital characteristics + county fixed effects		
	Point estimate	95% Wald confidence limits		Point estimate	95% Wald confidence limits		Point estimate	95% Wald confidence limits		Point estimate	95% Wald confidence limits	
Managed care	0.758	0.694	0.829	0.797	0.728	0.874	0.843	0.754	0.942	0.79	0.714	0.874
Age 18–44 years	0.801	0.688	0.933	0.802	0.689	0.934	0.776	0.661	0.911	0.806	0.688	0.943
Age 45–64 years	REF	REF	REF	REF	REF	REF	REF	REF	REF	REF	REF	REF
Age 65+ years	1.884	1.708	2.078	1.828	1.655	2.019	1.857	1.662	2.075	1.871	1.684	2.08
Male	REF	REF	REF	REF	REF	REF	REF	REF	REF	REF	REF	REF
Female	1.192	1.093	1.301	1.188	1.089	1.296	1.213	1.106	1.331	1.204	1.1	1.319
APRMORT_45002	17.472	11.606	26.303	17.306	11.494	26.059	18.513	12.194	28.107	17.421	11.539	26.301
APRMORT_45003	37.647	24.751	57.261	37.876	24.895	57.626	43.941	28.546	67.640	38.88	25.466	59.36
APRMORT_45004	286.246	190.76	429.527	297.971	198.442	447.421	429.277	281.752	654.044	327.875	217.389	494.516
APRMORT_44001	48.124	30.516	75.892	50.302	31.866	79.403	58.461	36.453	93.756	52.76	33.217	83.801
APRMORT_44002	31.378	20.633	47.719	32.77	21.531	49.876	38.932	25.279	59.958	33.019	21.609	50.454
APRMORT_44003	125.356	82.532	190.4	130.734	85.984	198.776	172.875	112.064	266.685	141.576	92.638	216.366
APRMORT_44004	>999.999	832.177	>999.999	>999.999	873.693	>999.999	>999.999	>999.999	>999.999	>999.999	>999.999	>999.999
APRMORT_21XXX	134.17	90.207	199.559	142.957	95.953	212.986	195.398	129.375	295.113	154.649	103.417	231.263
APRMORT_OTHER	49.024	32.526	73.89	51.902	34.385	78.342	68.715	44.962	105.018	53.797	35.508	81.505
Lowest income	0.973	0.855	1.107	0.925	0.811	1.055	0.929	0.791	1.092	0.996	0.846	1.174
Low income	1.028	0.912	1.158	0.959	0.848	1.084	0.993	0.859	1.148	1.044	0.9	1.212
Moderate income	1.041	0.93	1.166	1.01	0.901	1.132	1.063	0.932	1.212	1.065	0.934	1.214
High income	REF	REF	REF	REF	REF	REF	REF	REF	REF	REF	REF	REF
0-99 beds				1.417	1.118	1.796				1.35	1.032	1.766
100-299 beds				REF	REF	REF				REF	REF	REF
300-499 beds				0.899	0.792	1.021				0.885	0.767	1.021
500+ beds				0.96	0.829	1.111				0.885	0.749	1.045
Nonteaching				REF	REF	REF				REF	REF	REF
Teaching				0.983	0.872	1.108				1.003	0.87	1.156
Governmental				1.506	1.299	1.746				1.352	1.139	1.603
Not-for-profit				REF	REF	REF				REF	REF	REF
For-profit				0.832	0.678	1.022				0.976	0.764	1.247
Large metropolitan				REF	REF	REF						
Medium and small metropolitan				1.221	1.101	1.354						
Nonmetropolitan				1.423	1.141	1.774						

Abbreviation: *REF*, reference group

Notes: ^a^Patient characteristics were age, sex, All Patient Refined-Diagnosis Related Group (APR-DRG), and community income. ^b^Hospital characteristics were bed size, ownership, teaching status, and urban/rural location

Source: Agency for Healthcare Research and Quality, Center for Delivery, Organization, and Markets, Healthcare Cost and Utilization Project, State Inpatient Databases, 2009, from the following 11 states: Arizona, California, Connecticut, Massachusetts, Michigan, Minnesota, New Hampshire, Nevada, New York, Ohio, and Pennsylvania

**Table 9:** Association between Medicare managed care and inpatient mortality for pneumonia

Characteristic	Patient characteristics^a^			Patient + hospital characteristics^b^			Patient characteristic + hospital fixed effects			Patient + hospital characteristics + county fixed effects		
	Point estimate	95% Wald confidence limits		Point estimate	95% Wald confidence limits		Point estimate	95% Wald confidence limits		Point estimate	95% Wald confidence limits	
Managed care	1.032	0.982	1.085	1.072	1.019	1.128	0.989	0.932	1.050	1.047	0.992	1.105
Age 18–64 years	0.64	0.585	0.701	0.648	0.592	0.71	0.654	0.596	0.717	0.667	0.609	0.731
Age 65–74 years	REF	REF	REF	REF	REF	REF	REF	REF	REF	REF	REF	REF
Age 75–84 years	1.245	1.175	1.319	1.239	1.169	1.314	1.238	1.167	1.314	1.228	1.158	1.303
Age 85+ years	1.859	1.754	1.969	1.845	1.742	1.956	1.816	1.711	1.928	1.802	1.699	1.912
Male	REF	REF	REF	REF	REF	REF	REF	REF	REF	REF	REF	REF
Female	0.964	0.925	1.004	0.967	0.928	1.007	0.974	0.934	1.015	0.968	0.929	1.009
APRMORT_137xxx	24.16	18.166	32.131	24.55	18.459	32.652	28.237	21.203	37.605	27.485	20.65	36.58
APRMORT_139002	4.943	3.72	6.569	4.984	3.75	6.624	5.066	3.810	6.737	5.1	3.836	6.781
APRMORT_139003	19.988	15.087	26.481	20.675	15.605	27.394	22.743	17.150	30.161	22.143	16.703	29.35
APRMORT_139004	89.745	67.731	118.92	94.095	71.003	124.7	113.230	85.336	150.241	105.916	79.868	140.5
APRMORT_OTHER	118.202	89.115	156.78	126.055	95.007	167.25	139.748	105.195	185.649	135.944	102.388	180.5
Lowest income	0.963	0.907	1.022	0.913	0.858	0.972	0.920	0.847	0.999	0.956	0.884	1.034
Low income	0.97	0.917	1.026	0.908	0.857	0.963	0.978	0.908	1.054	0.984	0.916	1.058
Moderate income	0.942	0.891	0.996	0.923	0.872	0.976	0.999	0.934	1.068	0.989	0.928	1.054
High income	REF	REF	REF	REF	REF	REF	REF	REF	REF	REF	REF	REF
0-99 beds				1.15	1.075	1.23				1.269	1.174	1.372
100-299 beds				REF	REF	REF				REF	REF	REF
300-499 beds				0.942	0.892	0.995				0.968	0.909	1.03
500+ beds				0.987	0.917	1.062				0.948	0.873	1.03
Nonteaching				REF	REF	REF				REF	REF	REF
Teaching				0.882	0.834	0.933				0.903	0.844	0.967
Governmental				1.215	1.125	1.311				1.067	0.974	1.169
Not-for-profit				REF	REF	REF				REF	REF	REF
For-profit				1.051	0.973	1.135				1.048	0.956	1.148
Large metropolitan				REF	REF	REF						
Medium and small metropolitan				1.042	0.993	1.093						
Nonmetropolitan				1.17	1.085	1.263						

Abbreviation: *REF*, reference group

Notes: ^a^Patient characteristics were age, sex, All Patient Refined-Diagnosis Related Group (APR-DRG), and community income. ^b^Hospital characteristics were bed size, ownership, teaching status, and urban/rural location

Source: Agency for Healthcare Research and Quality, Center for Delivery, Organization, and Markets, Healthcare Cost and Utilization Project, State Inpatient Databases, 2009, from the following 11 states: Arizona, California, Connecticut, Massachusetts, Michigan, Minnesota, New Hampshire, Nevada, New York, Ohio, and Pennsylvania

**Table 10:** Association between private managed care and inpatient mortality for pneumonia

Characteristic	Patient characteristics^a^			Patient + hospital characteristics^b^			Patient characteristic + hospital fixed effects			Patient + hospital characteristics + county fixed effects		
	Point estimate	95% Wald confidence limits		Point estimate	95% Wald confidence limits		Point estimate	95% Wald confidence limits		Point estimate	95% Wald confidence limits	
Managed care	0.904	0.817	1.00	0.889	0.802	0.985	0.828	0.724	0.947	0.875	0.78	0.98
Age 18–44 years	0.396	0.33	0.476	0.393	0.328	0.472	0.374	0.308	0.454	0.393	0.326	0.474
Age 45–64 years	REF	REF	REF	REF	REF	REF	REF	REF	REF	REF	REF	REF
Age 65+ years	1.57	1.408	1.751	1.573	1.409	1.757	1.654	1.457	1.878	1.54	1.371	1.731
Male	REF	REF	REF	REF	REF	REF	REF	REF	REF	REF	REF	REF
Female	0.988	0.894	1.093	0.992	0.897	1.097	1.040	0.933	1.158	1.012	0.912	1.123
APRMORT_137xxx	88.389	46.656	167.452	88.596	46.759	167.866	109.154	57.155	208.459	101.242	53.279	192.383
APRMORT_139002	31.018	16.5	58.31	30.906	16.44	58.103	32.666	17.294	61.701	31.239	16.585	58.841
APRMORT_139003	140.387	75.185	262.133	140.697	75.342	262.744	172.269	91.576	324.066	155.064	82.832	290.284
APRMORT_139004	570.545	304.621	>999.999	576.589	307.753	>999.999	851.446	450.071	>999.999	687.987	365.93	>999.999
APRMORT_OTHER	517.875	277.751	965.593	518.855	278.119	967.97	669.828	355.990	>999.999	606.831	324.27	>999.999
Lowest income	0.811	0.697	0.943	0.832	0.712	0.972	0.870	0.714	1.060	0.937	0.775	1.134
Low income	0.798	0.696	0.914	0.822	0.713	0.947	0.917	0.769	1.093	0.89	0.751	1.055
Moderate income	0.911	0.801	1.035	0.931	0.818	1.06	1.014	0.870	1.181	1.009	0.872	1.168
High income	REF	REF	REF	REF	REF	REF	REF	REF	REF	REF	REF	REF
0-99 beds				1.214	1.018	1.447				1.2	0.971	1.483
100-299 beds				REF	REF	REF				REF	REF	REF
300-499 beds				1.012	0.883	1.159				1.026	0.879	1.198
500+ beds				0.892	0.749	1.062				0.851	0.699	1.035
Nonteaching				REF	REF	REF				REF	REF	REF
Teaching				1.221	1.063	1.402				1.204	1.021	1.419
Governmental				1.323	1.103	1.587				1.305	1.052	1.62
Not-for-profit				REF	REF	REF				REF	REF	REF
For-profit				0.868	0.699	1.079				0.87	0.68	1.113
Large metropolitan				REF	REF	REF						
Medium and small metropolitan				0.844	0.747	0.953						
Nonmetropolitan				0.879	0.718	1.077						

Abbreviation: *REF*, reference group

Notes: ^a^Patient characteristics were age, sex, All Patient Refined-Diagnosis Related Group (APR-DRG), and community income. ^b^Hospital characteristics were bed size, ownership, teaching status, and urban/rural location

Source: Agency for Healthcare Research and Quality, Center for Delivery, Organization, and Markets, Healthcare Cost and Utilization Project, State Inpatient Databases, 2009, from the following 11 states: Arizona, California, Connecticut, Massachusetts, Michigan, Minnesota, New Hampshire, Nevada, New York, Ohio, and Pennsylvania

**Table 11:** Association between Medicare managed care and inpatient mortality for congestive heart failure

Characteristic	Patient characteristics^a^			Patient + hospital characteristics^b^			Patient characteristic + hospital fixed effects		Patient + hospital characteristics + county fixed effects		
	Point estimate	95% Wald confidence limits		Point estimate	95% Wald confidence limits		Point estimate	95% Wald confidence limits	Point estimate	95% Wald confidence limits	
Managed care	0.950	0.904	0.998	0.981	0.933	1.031	Failed to converge		0.946	0.898	0.998
Age 18–64 years	1.009	0.908	1.122	1.028	0.925	1.143			1.058	0.951	1.177
Age 65–74 years	REF	REF	REF	REF	REF	REF			REF	REF	REF
Age 75–84 years	1.327	1.247	1.413	1.317	1.237	1.402			1.303	1.223	1.387
Age 85+ years	2.022	1.902	2.15	1.996	1.877	2.122			1.935	1.819	2.059
Male	REF	REF	REF	REF	REF	REF			REF	REF	REF
Female	0.896	0.86	0.933	0.898	0.861	0.935			0.89	0.854	0.928
APRMORT_161xxx	2.388	1.702	3.351	2.56	1.824	3.593			2.694	1.918	3.785
APRMORT_191xxx	7.093	5.329	9.442	7.573	5.687	10.084			8.173	6.133	10.892
APRMORT_194002	2.071	1.583	2.709	2.083	1.592	2.725			2.182	1.667	2.855
APRMORT_194003	7.415	5.69	9.663	7.613	5.842	9.922			8.285	6.354	10.803
APRMORT_194004	37.648	28.903	49.037	39.386	30.232	51.31			45.335	34.775	59.101
APRMORT_OTHER	15.041	11.432	19.789	16.007	12.162	21.066			17.347	13.171	22.846
Lowest income	0.881	0.83	0.935	0.832	0.782	0.885			0.828	0.766	0.894
Low income	0.971	0.919	1.027	0.91	0.859	0.965			0.942	0.878	1.012
Moderate income	0.96	0.908	1.014	0.943	0.891	0.997			1.008	0.946	1.074
High income	REF	REF	REF	REF	REF	REF			REF	REF	REF
0-99 beds				1.22	1.133	1.314			1.343	1.232	1.464
100-299 beds				REF	REF	REF			REF	REF	REF
300-499 beds				0.955	0.904	1.008			0.941	0.885	1.001
500+ beds				1.058	0.987	1.134			1.061	0.981	1.148
Nonteaching				REF	REF	REF			REF	REF	REF
Teaching				0.931	0.881	0.983			0.918	0.859	0.98
Governmental				1.137	1.046	1.237			1.012	0.919	1.115
Not-for-profit				REF	REF	REF			REF	REF	REF
For-profit				0.921	0.844	1.004			0.929	0.842	1.026
Large metropolitan				REF	REF	REF					
Medium and small metropolitan				1.005	0.958	1.054					
Nonmetropolitan				1.318	1.218	1.427					

Abbreviation: *REF*, reference group

Notes: ^a^Patient characteristics were age, sex, All Patient Refined-Diagnosis Related Group (APR-DRG), and community income. ^b^Hospital characteristics were bed size, ownership, teaching status, and urban/rural location

Source: Agency for Healthcare Research and Quality, Center for Delivery, Organization, and Markets, Healthcare Cost and Utilization Project, State Inpatient Databases, 2009, from the following 11 states: Arizona, California, Connecticut, Massachusetts, Michigan, Minnesota, New Hampshire, Nevada, New York, Ohio, and Pennsylvania

**Table 12:** Association between private managed care and inpatient mortality for congestive heart failure

Characteristic	Patient characteristics^a^			Patient + hospital characteristics^b^			Patient characteristic + hospital fixed effects		Patient + hospital characteristics + county fixed effects	
	Point estimate	95% Wald confidence limits		Point estimate	95% Wald confidence limits		Point estimate	95% Wald confidence limits	Point estimate	95% Wald confidence limits
Managed care	0.621	0.549	0.704	0.643	0.567	0.729	Failed to converge		Failed to converge	
Age 18–44 years	1.012	0.74	1.386	0.989	0.722	1.354				
Age 45–64 years	REF	REF	REF	REF	REF	REF				
Age 65+ years	2.036	1.785	2.322	2.131	1.865	2.435				
Male	REF	REF	REF	REF	REF	REF				
Female	1.165	1.035	1.312	1.172	1.041	1.32				
APRMORT_161xxx	2.649	1.686	4.162	2.498	1.586	3.932				
APRMORT_191xxx	2.739	1.803	4.162	2.612	1.716	3.975				
APRMORT_194002	2.32	1.613	3.337	2.32	1.613	3.337				
APRMORT_194003	5.392	3.775	7.703	5.366	3.756	7.666				
APRMORT_194004	28.071	19.641	40.12	28.013	19.592	40.053				
APRMORT_OTHER	9.41	6.374	13.894	8.99	6.078	13.298				
Lowest income	0.736	0.619	0.876	0.713	0.596	0.853				
Low income	0.82	0.699	0.961	0.829	0.703	0.977				
Moderate income	0.859	0.736	1.004	0.854	0.73	1.000				
High income	REF	REF	REF	REF	REF	REF				
0-99 beds				1.009	0.803	1.266				
100-299 beds				REF	REF	REF				
300-499 beds				0.983	0.834	1.158				
500+ beds				1.089	0.885	1.34				
Nonteaching				REF	REF	REF				
Teaching				1.144	0.967	1.354				
Governmental				1.601	1.289	1.988				
Not-for-profit				REF	REF	REF				
For-profit				0.716	0.53	0.966				
Large metropolitan				REF	REF	REF				
Medium and small metropolitan				1.121	0.975	1.289				
Nonmetropolitan				0.804	0.618	1.045				

Abbreviation: *REF*, reference group

Notes: ^a^Patient characteristics were age, sex, All Patient Refined-Diagnosis Related Group (APR-DRG), and community income. ^b^Hospital characteristics were bed size, ownership, teaching status, and urban/rural location

Source: Agency for Healthcare Research and Quality, Center for Delivery, Organization, and Markets, Healthcare Cost and Utilization Project, State Inpatient Databases, 2009, from the following 11 states: Arizona, California, Connecticut, Massachusetts, Michigan, Minnesota, New Hampshire, Nevada, New York, Ohio, and Pennsylvania

## Abbreviations

AHA: American Hospital Association
AMI: Acute myocardial infarction
APR-DRG: All Patient Refined Diagnosis-Related Group
CHF: Congestive heart failure
HCUP: Healthcare Cost and Utilization Project
HMO: Health maintenance organization
IQI: Inpatient Quality Indicator
SID: State Inpatient Databases

## References

[1] Andrews R, Russo CA, Pancholi M (2007). Trends in hospital risk-adjusted mortality for select diagnoses and procedures, 1994–2004. HCUP statistical brief #38.

[2] Hines A, Stranges E, Andrews RM (2010). Trends in hospital risk-adjusted mortality for select diagnoses by patient subgroups, 2000–2007. HCUP statistical brief #98.

[3] Hines AL, Heslin KC, Jiang HJ, Coffey R (2015). Trends in observed adult inpatient mortality for high-volume conditions, 2002–2012. HCUP statistical brief #194.

[4] Carlisle DM, Siu AL, Keeler EB, McGlynn EA, Kahn KL, Rubenstein LV (1992). HMO versus fee-for-service care of older persons with acute myocardial infarction. Am J Public Health.

[5] Smith MA, Frytak JR, Liou J, Finch MD (2005). Rehospitalization and survival for stroke patients in managed care and traditional medicare plans. Med Care.

[6] Afendulis CC, Chernew ME, Kessler DP (2013). The effect of managed care advantage on hospital admissions and mortality.

[7] Angus DC, Linde-Zwirble WT, Sirio CA, Rotondi AJ, Chelluri L, Newbold RC (1996). The effect of managed care on ICU length of stay: implications for medicare. JAMA.

[8] Schnitzler MA, Lambert DL, Mundy LM, Woodward RS (1998). Variations in healthcare measures by insurance status for patients receiving ventilator support. Clin Perform Qual Health Care.

[9] Bennett KM, Scarborough JE, Pappas TN, Kepler TB (2010). Patient socioeconomic status is an independent predictor of operative mortality. Ann Surg.

[10] Ingram DD, Franco SJ. NCHS urban–rural classification scheme for counties. National Center for Health Statistics. Vital Health Stat. 2012;2(154):1–65. 22783637

[11] Dowd B, Maciejewski ML, O’Connor H, Riley G, Geng Y (2011). Health plan enrollment and mortality in the medicare program. Health Econ.

[12] Agency for Healthcare Research and Quality (2009). Inpatient quality indicators: software documentation. Version 4.1 SAS.

[13] Friedman B, Jiang HJ (2010). Do medicare advantage enrollees tend to be admitted to hospitals with better or worse outcomes compared with fee-for-service enrollees?. Int J Health Care Finance Econ.

[14] Ricketts TC (2000). The changing nature of rural health care. Annu Rev Public Health.

[15] Maeng DD, Martsolf GR (2011). Comparing patient outcomes across payer types: implications for using hospital discharge records to assess quality. Health Serv Res.

